# Sensor Fusion-Based Approach to Eliminating Moving Objects for SLAM in Dynamic Environments

**DOI:** 10.3390/s21010230

**Published:** 2021-01-01

**Authors:** Xiangwei Dang, Zheng Rong, Xingdong Liang

**Affiliations:** 1 National Key Laboratory of Microwave Imaging Technology, Aerospace Information Research Institute, Chinese Academy of Sciences, Beijing 100094, China; dangxiangwei16@mails.ucas.ac.cn; 2 School of Electronic, Electrical and Communication Engineering, University of Chinese Academy of Sciences, Beijing 100049, China; 3 National Laboratory of Pattern Recognition, Institute of Automation, Chinese Academy of Sciences, Beijing 100190, China; zheng.rong@nlpr.ia.ac.cn

**Keywords:** SLAM, dynamic environments, LiDAR, mmW-radar, sensor fusion, moving objects

## Abstract

Accurate localization and reliable mapping is essential for autonomous navigation of robots. As one of the core technologies for autonomous navigation, Simultaneous Localization and Mapping (SLAM) has attracted widespread attention in recent decades. Based on vision or LiDAR sensors, great efforts have been devoted to achieving real-time SLAM that can support a robot’s state estimation. However, most of the mature SLAM methods generally work under the assumption that the environment is static, while in dynamic environments they will yield degenerate performance or even fail. In this paper, first we quantitatively evaluate the performance of the state-of-the-art LiDAR-based SLAMs taking into account different pattens of moving objects in the environment. Through semi-physical simulation, we observed that the shape, size, and distribution of moving objects all can impact the performance of SLAM significantly, and obtained instructive investigation results by quantitative comparison between LOAM and LeGO-LOAM. Secondly, based on the above investigation, a novel approach named EMO to eliminating the moving objects for SLAM fusing LiDAR and mmW-radar is proposed, towards improving the accuracy and robustness of state estimation. The method fully uses the advantages of different characteristics of two sensors to realize the fusion of sensor information with two different resolutions. The moving objects can be efficiently detected based on Doppler effect by radar, accurately segmented and localized by LiDAR, then filtered out from the point clouds through data association and accurate synchronized in time and space. Finally, the point clouds representing the static environment are used as the input of SLAM. The proposed approach is evaluated through experiments using both semi-physical simulation and real-world datasets. The results demonstrate the effectiveness of the method at improving SLAM performance in accuracy (decrease by 30% at least in absolute position error) and robustness in dynamic environments.

## 1. Introduction

In recent decades, autonomous robots widely used in various fields such as urban warfare, rescue after disaster, autonomous driving and space robotics have attracted more and more attention. A crucial characteristic of an autonomous mobile robot is its ability to determine its whereabouts and make sense of its surrounding environments [1]. Simultaneous Localization and Mapping (SLAM) is a prerequisite for many robotic applications, which involves a system that simultaneously completes the positioning of the mobile robot itself and the map construction of the surrounding environment without any prior information [2]. Therefore, SLAM has been vigorously pursued in the mobile robot research field and various excellent algorithms have emerged. Generally, they can be divided into vision-based SLAM and LiDAR-based SLAM. Visual SLAM such as MonoSLAM [3], DSO [4], ORB-SLAM [5] and VINS-Mono [6], where the primary sensor is the camera, can get rich information from the environment, but they are sensitive to texture richness and illumination. In contrast, LiDAR-based SLAM is widely used because it can acquire accurate and reliable distance information from the surrounding environment for state estimation. LiDAR-SLAM can be further divided into filter-based and optimization-based method, such as Gmapping [7], Hector SLAM [8], LOAM [9] and Cartographer [10] etc.

However, most of the popular LiDAR-SLAM frameworks deem the environment as motionless and ignore the influence of moving objects, while the assumption of the static environment can be easily violated in the real world, which will result in degradation of localization and mapping performance [11]. Due to the existence of dynamic objects, unexpected features will be extracted and used in the inter-frame matching and back-end optimization, which can introduce estimation error of robots’ pose and surrounding map. Taking space robots as an example, in orbital or on planetary environments, the robots may cooperate with other agents and astronauts and encounter floating objects, which forms a challenging dynamic scenario.

To address this issue, SLAM algorithms specially for dynamic scenarios have been proposed. Some of these methods try to implicitly reduce the influence of dynamic objects using filtering or probability-based techniques [12]. The other methods explicitly detect the moving objects through various methods such as optical flow [13], deep learning [14,15,16,17], and then eliminate them from the raw sensor measurements to guarantee the accuracy of perception. However, these methods can only deal with the scenario with a few moving objects in good environmental conditions, otherwise the performance will be degraded or even fail.

In this paper, we first propose a semi-physical simulation method to quantitatively analyze the impact of moving objects on LiDAR-based SLAM performance in various dynamic scenarios. This method models the moving objects in the environment and generates echo according to the measurement model of LiDAR, and then integrates the measurements with the data measured in the real scene to achieve semi-physically simulated datasets in dynamic scenarios. We quantitatively evaluate the influence of the shape, quantity, speed, and distribution of the moving objects on the performance of LiDAR-based SLAM, towards providing theoretical background for the further proposed method of eliminating moving objects for SLAM.

Secondly, we propose a novel approach to eliminating the influence of dynamic objects named EMO (eliminating the moving objects) to improve the performance of SLAM by fusing mmW-radar and LiDAR. The LiDAR has a wide field of view (FOV) and can provide accurate distance and angle measurements with high resolution, but it is not straight forward to detect dynamic objects. On the opposite, mmW-radar can easily and directly provide velocity measurement of the objects using the Doppler effect, but it has a narrow FOV and low angular resolution. The fusion of the data from both sensors can thus benefit from their complementary [18]. The proposed method can effectively fuse the information from both sensors and precisely recognize the moving objects. The point clouds from LiDAR are segmented and at the same time the ghost targets in mmW-radar measurements are rejected by verification using LiDAR measurements. Then the segmented point clouds and mmW-radar measurements are associated with yield the precise volume and location of the moving objects. The actually moving objects are finally determined by compensating the Doppler-velocity from mmW-radar with sensor’s velocity from real-time SLAM feedback, and eliminated from original point clouds. The filtered point clouds are used as the input of SLAM.

The proposed method is demonstrated by semi-physical simulation and experiments in the real world. The results show that the moving objects can be efficiently and precisely identified and rejected from point clouds in complex environment, which significantly improve the accuracy and robustness of the LiDAR-based SLAM. The main contributions of this paper are: (1) An effective semi-physical simulation method is proposed to analyze the impact of dynamic scenarios on SLAM performance, taking into account the size, distribution, shape and speed of the objects. The quantitative results provide us a solid theoretical basis for the subsequent proposal of the moving objects elimination method. (2) A moving objects elimination method (EMO) is proposed to improve the performance of SLAM. This method effectively takes advantages of the mmW-radar and LiDAR to realize real-time detection, verification, and rejection of moving objects in environments for the purpose of improving the accuracy and robustness of localization and mapping. (3) The first system containing LiDAR and radar for moving target removal is built. Based on the methods of temporal synchronization and spatial calibration designed by us, the system can fuse the information of the two sensors at the front end to remove the dynamic target in real time, and then uses the filtered point cloud as the input of LiDAR-SLAM to improve the overall performance. (4) The proposed method is fully evaluated using semi-physically simulated datasets and real-world datasets in various scenarios to demonstrate the effectiveness and efficiency of the method.

The rest of the paper is organized as follows: Section 2 reviews related works. Section 3 introduces our method in detail, including semi-physical simulation and moving objects elimination. Section 4 shows the experiments details and results. Finally, Section 5 concludes our work.

## 2. Related Works

It is well acknowledged that the existence of moving objects in environments can impact the performance of SLAM to some degree. However, there are only a few pieces of research work on the evaluation and investigation of this influence. Pancham et al. [19] evaluate the performance of ORB-SLAM with an RGB-D camera in a dynamic environment including an object moving at a range of specific linear speeds. Experiments show that a moving object at lower speeds degrades the performance of ORB-SLAM, and removing the moving object can improve the performance of ORB-SLAM. Lu Z et al. [20] describe and compare three different approaches of SLAM in dynamic outdoor environments. They proposed a probabilistic SLAM with random sampling consensus(RANSAC) estimation, which shows better performance in the noisy environment to build the occupancy grid maps in their experiment. Roesler O et al. [21] evaluate four different 2D SLAM algorithms available in Robot Operating System (ROS) and find that Hector Mapping achieves the best performance in dynamic scenarios. These studies evaluate the performance of SLAM in dynamic environments, but most of them simply compare some algorithms by building simple scenarios. The correlation and quantitative analysis between dynamic objects and SLAM performance are not given. This is mainly because the data collection of datasets in various dynamic scenarios is difficult, time-consuming and laborious, while semi-physical simulation can solve this problem.

To mitigate the influence of dynamic objects on the SLAM performance, a variety of SLAM algorithms in dynamic environment have been developed. These methods can be generally divided into two categories. The first category of methods does not detect the dynamic objects directly, but implicitly reduces the influence of moving objects using filtering or probability-based techniques. The second category of methods try to explicitly recognize the moving objects using methods such as optical flow, deep learning [14,15,16,17], and then eliminates them to guarantee the accuracy of estimation.

Kitt et al. [22] and Tan et al. [23] propose to use RANSAC method to eliminate mismatched information caused by moving objects to improve odometer accuracy. Hhnel D et al. [24] present a new approach that interleaves mapping and localization with probabilistic technique to identify spurious measurements. Bibby C et al. [25] combine the least-squares formulation of SLAM and sliding window optimization together with generalized expectation maximization, to incorporate both dynamic and stationary objects directly into SLAM estimation. Probabilistic grid map [12] is used to decrease the influence of dynamic objects on map construction. Huber norms are also widely used during optimization to mitigate the degradation caused by the dynamic outliers. However, these methods will fail in the scenario with many dynamic objects because they are not able to explicitly recognize the moving objects.

Some dynamic SLAM algorithms based on dynamic objects detection are also proposed [13,26,27,28,29], trying to precisely detect and reject the dynamic objects to improve the performance of SLAM system, while moving objects detection under dynamic background becomes a new challenge. X Zhang et al. [1] incorporate the sensor information of a monocular camera and laser range finder to remove the feature outliers related to dynamic objects to enhances the accuracy of the localization. Wangsiripitak and Murray [30] try to reject moving outliers by tracking known 3D dynamic objects. Moo et al. [31] use two single Gaussian models that can effectively represent the foreground and background to find the moving targets. Sun et al. [32] extended the concept of intensity difference image to identify the boundaries of dynamic objects, and use a motion removal approach as a preprocessing stage to filter out the data associated with moving objects. Zhao H et al. [33] use GPS data and control inputs to diagnose pose error and classify objects with moving object detection and tracking.

Deep learning [14,15,16,17]-based methods are also used to detect the object for SLAM recently. Advanced convolutional neural network (CNN) architectures such as YOLO [34], SSD ( Single Shot multibox Detector ) [35], SegNet [36], and Mask R-CNN [37] can effectively detect the labels of the objects in a scene. Han and Xi [38] propose PSPNet-SLAM (Pyramid Scene Parsing Network-SLAM) to improve ORB-SLAM2, where PSPNet and the optical flow are used to detect dynamic features. DS-SLAM [14] combines semantic segmentation network with moving consistency checking method to reduce the impact of dynamic objects, and thus improve the localization accuracy in dynamic environments. DynaSLAM [15], built on ORB-SLAM2, has the capabilities of dynamic object detection and background inpainting, and the resulting accuracy outperforms the standard visual SLAM baselines in highly dynamic scenarios. Semantic information is also used to detect the dynamic objects. Yang S et al. [16] present a semantic and geometric constrained method SGC-VSLAM, which is built on the RGB-D version of ORB-SLAM2 with the addition of dynamic object detection and static map construction to filter out outliers. Xieyuanli Chen et al. [17] propose SuMa++ to reduce the influence of moving objects. By directly performing semantic segmentation on the 3D point cloud, potential dynamic targets in the environment, such as cars, etc., are recognized and removed to construct a static semantic map. Henein M et al. [39] propose a new feature-based, model-free, object-aware dynamic SLAM algorithm that exploits semantic segmentation to enable robust estimation of motion of robot. However, the above learning-based dynamic detection methods have some drawback. These methods cannot be generalized to all kinds of moving objects in the real world, and the detected dynamic targets are not necessarily moving actually [40]. They also require a lot of training work, and consume considerable computation to achieve real-time performance. Furthermore, most of these methods are based on LiDAR or vision, and will fail in challenging environments such as poor lighting, rain and fog, etc. However, our method (EMO) directly obtains the real moving target in the environment through the measurement of mmW-radar, without semantic cues or prior knowledge and is suitable for any unknown rigid objects. To the best of our knowledge, this is the first dynamic SLAM system that combines of radar and LiDAR for moving targets removal.

## 3. Methodology

In this section, we first present the method of quantitative analysis to investigating the impact of objects with different dynamic patterns on SLAM performance through semi-physical simulation. Secondly, we propose an effective method to eliminating the influence of moving objects (EMO) on SLAM based on multi-sensor fusion. The investigation result in the first part provides a solid theoretical and empirical background knowledge for the second part of work.

### 3.1. Semi-Physical Simulation for Evaluation of LiDAR-Based SLAM

**Figure 1:**
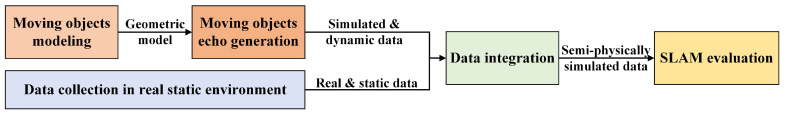
Semi-physical simulation-based LiDAR-SLAM evaluation.

To precisely investigate the influencing factors of dynamic environments on the performance of SLAM, datasets in the environments with various dynamic patterns must be collected. Semi-physical simulation is used in this work to precisely control the appearance of moving objects in the environment. First, typical moving objects in the real world are modeled as two types of elements with specified size, shape, quantity and motion model; then according to the measurement model of LiDAR used in the experiment (here is VLP-16 or HDL-64), the echo data of simulated objects is generated and integrated with the data corresponding to the static environment collected in the real world. We use these synthetic datasets to evaluate the state-of-the-art SLAM algorithms and our methods. The semi-physical simulation is depicted in Figure 1.

#### 3.1.1. Moving Objects Modeling

**Figure 2:**
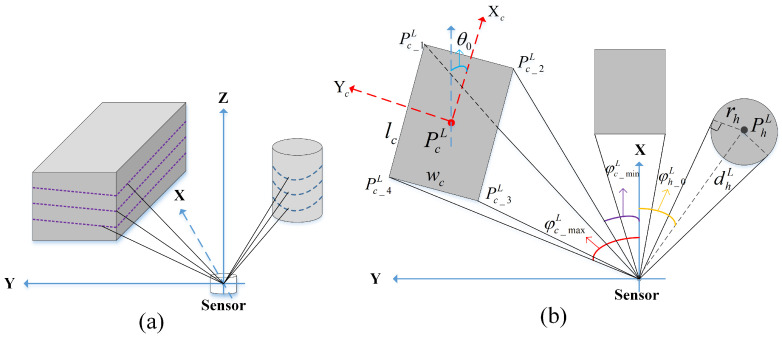
The geometric models used for dynamic objects simulation, cuboids and cylinder, are scanned by LiDAR (**a**). According to their poses $P_{c}^{L}\left(x_{o},y_{o},\theta_{o}\right)$ and $P_{h}^{L}\left(x_{o},y_{o}\right)$ in the sensor coordinate frame, the LiDAR measurements can be simulated respectively (**b**), which is calculated in XY plane of the sensor frame. We denote the world coordinate frame as *W*, the sensor coordinate frame as *L*, and the robot body frame as *B*. Depending on the position and shape of the object, one or two sides of the object can be detected. By evaluating the boundary of the models in sensor’s FOV the echo data can be calculated.

In different robot application areas, the type of dynamic objects in the environment is different, but in most scenarios, the moving objects are mainly vehicles and pedestrians. Here, we use cuboids with specified length, width and height to simulate vehicle in the environment and cylinders to simulate pedestrians. The reason we choose two different geometric shapes is that we found different SLAM algorithms are sensitive to different geometric information because they use different methods to do the frame matching. The SLAM algorithms such as LOAM and LeGO-LOAM are feature-based methods, where the feature is extracted by evaluating the curvature of the point and are sensitive to the existence of cuboids. However, algorithm such as Cartographer use grid map-based method, in which all the points are considered. Without losing generality and rationality for the evaluation, we use cuboids and cylinder in our simulation, as shown in Figure 2a.

We denote the world coordinate frame as *W*, the sensor coordinate frame as *L*, and the robot body frame as *B*. We assume that the pose of the cube in the sensor coordinate frame is $P_{c}^{L}\left(x_{o},y_{o},z_{o},\theta_{o}\right)$, where $\theta_{o}$ is the heading of the cuboid, i.e., the angle between the x-axis of cuboid and the x-axis of sensor, as shown in Figure 2b. Similarly, the center of the cylinder represented in sensor frame is denoted as $P_{h}^{L}\left(x_{o},y_{o},z_{o}\right)$.

Since the goal of simulation is to get the echo of moving objects measured by 3D LiDAR, for the simplicity of representation and implementation, we analyze the echo of a single-channel LiDAR first. We project the cuboid and cylinder onto the XY plane of the sensor frame, yielding a rectangle and a circle. The length and width of rectangle are $l_{c}$ and $w_{c}$, and its center point is $P_{c}^{L}\left(x_{o},y_{o},\theta_{o}\right)$. The radius of the circle is $r_{h}$, and its center is $P_{h}^{L}\left(x_{o},y_{o}\right)$.

To correctly generate the sensor echo from simulated objects, the boundary of the models in sensor’s FOV need to be determined according to their poses in the sensor coordinate frame, as shown in Figure 2b. For a rectangle, according to the specified center point $P_{c}^{L}$ and heading angle $\theta_{o}$, its four vertices $P_{c\_1}^{L}\left(x_{1},y_{1}\right)$, $P_{c\_2}^{L}\left(x_{2},y_{2}\right)$,$P_{c\_3}^{L}\left(x_{3},y_{3}\right)$, $P_{c\_4}^{L}\left(x_{4},y_{4}\right)$ can be determined by Equation (1). Then the angle of each vertex with respect to the sensor frame and their maximum $\varphi_{c\_\max}^{L}$ and minimum $\varphi_{c\_\min}^{L}$ can be determined, consequently the corresponding vertices $P_{c\_\min}^{L}\left(x_{\min},y_{\min}\right)$, $P_{c\_\max}^{L}\left(x_{\max},y_{\max}\right)$ can be used to determine the boundary of the object observed by the sensor in the current pose. In particular, if two sides of the object can be seen, we also need to find the middle vertex $P_{c\_mid}^{L}\left(x_{mid},y_{mid}\right)$, and then the echo data can be calculated by Equations (2) and (3). Finally, according to the vertical resolution and FOV of the LiDAR, the echo data can be easily extended to 3D.

(1) $$\begin{array}{c} \left(x_{1},y_{1}\right)=\left(x_{o}+\frac{w_{c}}{2}\sin\theta_{o}+\frac{l_{c}}{2}\cos\theta_{o},y_{o}+\frac{w_{c}}{2}\cos\theta_{o}-\frac{l_{c}}{2}\sin\theta_{o}\right)\\ \left(x_{2},y_{2}\right)=\left(x_{o}-\frac{w_{c}}{2}\sin\theta_{o}+\frac{l_{c}}{2}\cos\theta_{o},y_{o}-\frac{w_{c}}{2}\cos\theta_{o}-\frac{l_{c}}{2}\sin\theta_{o}\right)\\ \left(x_{3},y_{3}\right)=\left(x_{o}-\frac{w_{c}}{2}\sin\theta_{o}-\frac{l_{c}}{2}\cos\theta_{o},y_{o}-\frac{w_{c}}{2}\cos\theta_{o}+\frac{l_{c}}{2}\sin\theta_{o}\right)\\ \left(x_{4},y_{4}\right)=\left(x_{o}+\frac{w_{c}}{2}\sin\theta_{o}-\frac{l_{c}}{2}\cos\theta_{o},y_{o}+\frac{w_{c}}{2}\cos\theta_{o}+\frac{l_{c}}{2}\sin\theta_{o}\right) \end{array}$$

(2) $$\left\{\begin{array}{c} \left(y_{\max}-y_{\min}\right)x+\left(x_{\min}-x_{\max}\right)y+x_{\max}y_{\min}-x_{\min}y_{\max}=0\\ y=kx,k\in \left(\tan\varphi_{c\_\min}^{L},\tan\varphi_{c\_\max}^{L}\right) \end{array}\right.$$

(3) x=xminymax−xmaxyminxminymax−xmaxyminymax−ymin+kxmin−kxmaxymax−ymin+kxmin−kxmax,k∈tanφc_minL,tanφc_maxLy=kx=kxminymax−xmaxyminkxminymax−xmaxyminymax−ymin+kxmin−kxmaxymax−ymin+kxmin−kxmax

For a circle, according to the coordinates of circle center, the distance $d_{h}^{L}$ and angle $\varphi_{h\_o}^{L}$ of the center point in sensor frame can be determined, and thus the echo occupied by the circle can be calculated using Equations (4) and (5). This equation has two sets of solutions, and we choose the solution set with the smallest Euclidean distance.

(4) $$\left\{\begin{array}{c} {\left(x-x_{o}\right)}^{2}+{\left(y-y_{o}\right)}^{2}=r_{h}^{2}\\ y=kx,k\in \left(\tan\left(\varphi_{h\_o}^{L}-a\tan\frac{r_{h}}{d_{h}^{L}}\right),\tan\left(\varphi_{h\_o}^{L}+a\tan\frac{r_{h}}{d_{h}^{L}}\right)\right) \end{array}\right.$$

(5) $$\left\{\begin{array}{c} x=\frac{2\left(x_{o}+ky_{o}\right)\pm \sqrt{4{\left(x_{o}+ky_{o}\right)}^{2}-4\left(1+k^{2}\right)\left(x_{o}^{2}+y_{o}^{2}-r_{h}^{2}\right)}}{2\left(1+k^{2}\right)},\\ y=kx=\frac{2k\left(x_{o}+ky_{o}\right)\pm k\sqrt{4{\left(x_{o}+ky_{o}\right)}^{2}-4\left(1+k^{2}\right)\left(x_{o}^{2}+y_{o}^{2}-r_{h}^{2}\right)}}{2\left(1+k^{2}\right)}\\ k\in \left(\tan\left(\varphi_{h\_o}^{L}-a\tan\frac{r_{h}}{d_{h}^{L}}\right),\tan\left(\varphi_{h\_o}^{L}+a\tan\frac{r_{h}}{d_{h}^{L}}\right)\right) \end{array}\right.$$

As mmW-radar is used in the elimination stage, the simulation of the radar is also required in the datasets. First, we assume that the radar data has been synchronized and calibrated. Due to the position of the simulated moving object is known when we generate the LiDAR dataset, accordingly we can use the centroid of the object as the echo point from radar, represent with the object’s distance, angle and speed. Considering the ranging accuracy of the radar, we also add random noise to the radar data for realistic simulation.

#### 3.1.2. Datasets Generation

After modeling the single moving object, in this subsection we detail the generation of semi-physically simulated datasets by simulating multiple objects with specified motion models and integrating the echo data with real static data.

**Figure 3:**
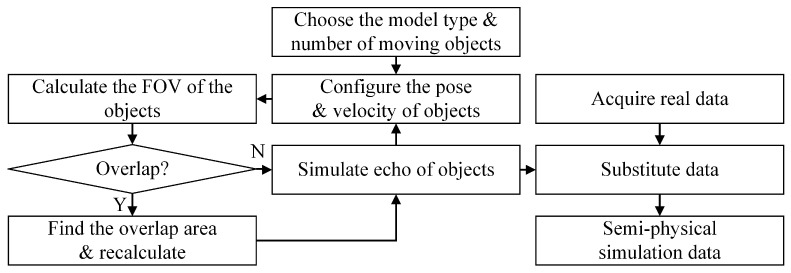
Generation of semi-physically simulated datasets. The real measurement data is partially substituted by the echo data from simulated moving objects.

Based on the knowledge of dynamics and kinematics [41], we specify a motion pattern for each single moving object, including the initial pose, linear and angular velocity, then the pose of each object at any time point can be obtained. In particular, we deal with the problem of mutual occlusion between the multiple moving targets, towards getting the simulated datasets consistent with the actual sensor measurement model. We evaluate the distance and occupied FOV of all the objects in the sensor frame, and try to find the overlaps among the objects. If any coincidence is found, we recalculate the echo data in the sensor FOV with occlusion. To integrate the simulated data with the real measurements, the sensor FOV occupied by the moving objects is calculated, and the real measurements in this area are substituted using the simulated data. By far we complete the semi-physical simulation of one frame. The complete datasets can be synthesized in this manner frame by frame. The process can be depicted as Figure 3.

### 3.2. Moving Objects Elimination for LiDAR-SLAM

**Figure 4:**
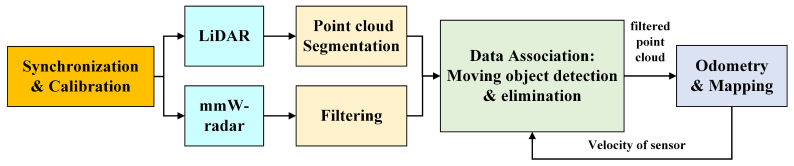
The system overview of moving objects elimination.

The existence of moving objects in the environment will corrupt the assumption of static environment for SLAM, which will significantly degrade the performance of localization and mapping. We propose a method using multi-sensor fusion to eliminate the influence of dynamic environments on the accuracy and robustness of robot state estimation. The system diagram is depicted in Figure 4.

First, temporal synchronization and spatial calibration [42] between LiDAR and mmW-radar are requisite step for accurate multi-sensor fusion. For timing synchronization, we use an ARM-based embedded hardware system to precisely synchronize the clock of LiDAR and mmW-radar. The hardware system is based on STM32F103 which uses the Cortex-M3 core, with a maximum CPU speed of 72 MHz. The hardware system will generate Pulse Per Second (PPS) signal to the LiDAR, and at the same time, the processor reads the radar data from the CAN bus to ensure that the LiDAR and radar data are measured simultaneously. For spatial calibration, due to the limited measurement accuracy and low resolution of the mmW-radar, we directly use CAD data to determine the relative translation and rotation between two sensors. Since the angular resolution and ranging accuracy of the radar are far lower than that of the LiDAR, the method based on CAD design and careful mounting can meet the accuracy requirement of sensor fusion of the two sensors.

After having two kinds of sensor data synchronized in time and space, we preprocess the measurement data from LiDAR and mmW-radar, mainly including the segmentation of LiDAR point clouds to identify objects and filtering of mmW-radar detection to reject ghost targets. Then we associate the preprocessed data from two sensors. Based on the measurement of Doppler effect by radar and velocity feedback from SLAM, we can precisely determine the actually moving objects with reference to the world. At the same time, the precise volume and location of the targets can be obtained by associating the LiDAR measurements. Finally the detected moving objects can be rejected from point clouds and these filtered data are used as the input of odometry and mapping modules in the LiDAR-based SLAM framework.

#### 3.2.1. Point Cloud Segmentation

Since the LiDAR provides dense point clouds representing the surroundings without any texture and clustering information, it is necessary to segment the point clouds first for fusing LiDAR measurements and the targets detected by mmW-radar, i.e., finding the correspondence between point clusters and radar targets.

In the segmentation, we first find the ground plane from point clouds using the similar method with [43]. Then an image-based segmentation method [44] is used to identify and separate the objects in the point clouds. The point clouds are organized into a range image with the size of vertical points number multiplied by LiDAR channel number. Whether a cluster of LiDAR points are corresponded to the same object can be determined by checking the angle between two vectors on the range image, and the points from the same object will be labeled identically.

**Figure 5:**
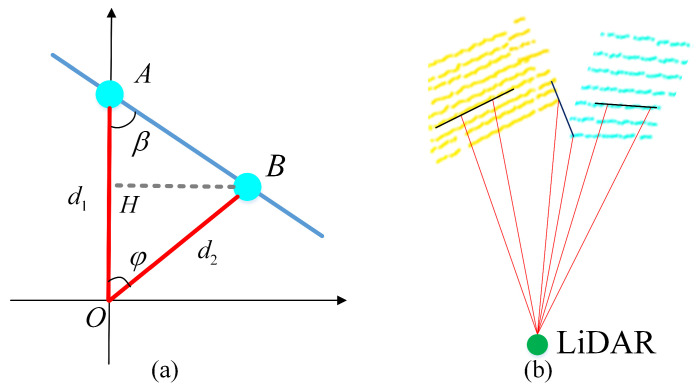
The schematic diagram of segmentation. (**a**) Whether two points are corresponded to the same object can be determined by evaluating the angle β. (**b**) shows an example.

The point clouds are projected onto a distance image first. As shown in Figure 5, *A* and *B* are two adjacent points on the distance image, OA and OB are distance vector directly measured by the LiDAR, and φ is the angle between these two vectors, which can be calculated according to the horizontal and vertical resolution and the position of two points in the image. Then we can calculate the angle β using Equation (6), and determine whether the two points are from the same object: the smaller the angle β and the greater the distance ∥AB∥ between the two points, the less likely the two points are from the same object. By setting the threshold of the angle (10 degrees in our test), the point clouds corresponding to the same object can be identified. For example, in Figure 5b, though the distance in the three sets of evaluated point-pair is similar, we can still correctly cluster the point clouds by furtherly checking the angle β between the two points. This segmentation approach is computing efficient and easy to implement, compared with the learning-based method. A failure case can be a situation in which the scanned object is planar, such as a wall, and oriented nearly parallel to the laser beams which result in an over-segmentation. Despite this shortcoming, our experiments suggest that the method is effective in practice. The aforementioned scenes occur rarely and if so, it usually results only in an over-segmentation of particularly inclined planar objects.

(6) $$\beta \;=\;\mathrm{atan}2\left(∥BH∥,∥HA∥\right)=\mathrm{atan}2\left(d_{2}\sin\varphi ,d_{1}-d_{2}\cos\varphi \right)$$

#### 3.2.2. Radar Results Filtering

Due to the propagation characteristics and multipath effect of electromagnetic waves, the detection results from mmW-radar are usually polluted by some non-target noise. To remove the negative impact of the false alarm, a two-stage filtering method is proposed.

In the preliminary filtering state, we use the valid flag included in the data frame of mmW-radar to remove invalid measurements. At the same time, according to the performance characteristics of the radar, some thresholds are set in terms of detection range, angle and speed to eliminate singular values.

**Figure 6:**
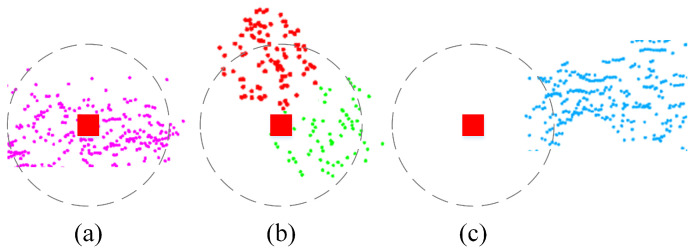
Radar measurement filtering using point cloud-based verification. (**a**) The mmW-radar target (red square) is valid as there are enough LiDAR points around it and the centroid of the segment is close enough to the radar target. (**b**) Two segmented objects are considered corresponding to the radar target. (**c**) Ghost target as the LiDAR point number in the specified radius is small and the point cluster is far from the target.

However, some ghost targets cannot be removed by the pre-filtering. Benefiting from the accurate measurements of surrounding environment from LiDAR, a verification strategy is furtherly proposed to identify the ghost targets. Based on the sensor calibration, the radar target points can be transformed to the LiDAR coordinate frame. As shown in Figure 6, a KD tree is built from point clouds, and we try to search K nearest neighbors around each radar point within a preset radius (which is determined by ranging accuracy of the radar. 0.5 m was used in this paper.) and compute the centroid of all the LiDAR points with the same label from the segmentation results. The radar point is valid only if the neighboring point number is above a threshold and the distance between radar point and the centroid is small enough, otherwise considered to be a ghost target. This method can effectively make use of the advantages of accurate target detection of LiDAR and effectively remove the radar false targets caused by multipath effect.

Through the two-stage filtering, the invalid measurements and false targets from the mmW-radar can be effectively reduced, which provides precise and reliable data for the following data association procedure.

#### 3.2.3. Data Association

The mmW-radar can directly obtain the velocity information of the detected targets in the environment, while LiDAR can obtain the accurate distance and direction information of the surrounding. By associating the data, dynamic targets in the environment can be accurately detected, identified and removed from point clouds to reduce the impact of moving objects on SLAM.

The velocity measurement of mmw-radar is mainly based on the Doppler effect, i.e., if the detected target is relatively moving with respect to the radar, the frequency of echo wave will be different from the frequency of the emitted wave. By detecting this frequency difference, the relative moving speed of the target can be calculated according to Equation (7).

(7) $$v=\frac{cf_{\Delta }}{2f_{o}}$$

where $f_{o}$ is the working frequency of mmW-radar, *c* is the speed of light, and $f_{\Delta }$ is the frequency difference measured by radar. Then the moving speed of the target relative to the radar can be obtained as *v*.

Since the mmW-radar can measure the relative speed of the target only in radial direction with reference to the moving sensors, motion compensation must be performed to get the target velocity in world frame and then identify the actually moving targets.

For each target, the Doppler-velocity $v_{tg}^{L}$ is measured by mmW-radar. For simplicity of expression, we assume that LiDAR and mmW-radar are in the same coordinate frame based on the aforementioned sensor calibration. By integrating the sensor velocity $v_{L}^{W}$ from SLAM feedback, the absolute velocity $v_{tg}^{sta}$ of the target in the current static frame can be estimated as

(8) $$v_{tg}^{sta}=R_{W}^{L}v_{L}^{W}+v_{tg}^{L}$$

where $R_{W}^{L}$ is inverse of the current orientation of the sensor in world frame. Thus, the actual moving status of the target can be determined.

Due to the limitation of mmW-radar measurements in resolution, density, accuracy, etc., the targets obtained by the radar can only be expressed as a few of sparse points in 2D, whereas the LiDAR can provide a bunch of accurate 3D dense points to represent the target. Thus, finding the accurate correspondence between sparse radar points and LiDAR point clouds is critical for the data fusion. We propose a data association algorithm based on 3D-to-2D projection and K neighborhood searching, as shown in Algorithm 1. In particular, the moving targets in our application mainly move on the ground plane, such as vehicles and pedestrians, so we assume that there can only be one target in any specified x-y 2D position, which makes it reasonable to fuse two-dimensional mmW-radar data with three-dimensional LiDAR data.
**Algorithm 1** Data Association**Input**    $P^{seg}$, $P^{rad}$, *C*, *D* **Output**
$P^{sta}$ 1: $P^{seg}$ is projected onto radar plane $\to P^{seg^{\prime }}$ 2: $P^{seg^{\prime }}$→ KD tree 3: **for**
$p_{j}^{rad}\in P^{rad}$
**do** 4:     KNN search( $P^{seg^{\prime }},p_{j}^{rad},R_{search}$ ) $\to P^{nb}$ 5:     **for**
$P_{k}^{nb}\in P^{nb}$
**do** 6:        Count point number in $P_{k}^{nb}\to c$ 7:        Compute centroid of all points in $P_{k}^{seg^{\prime }}\to e_{k}$ 8:        Compute distance between $p_{j}^{rad}$ and $e_{k}\to d$ 9:        **if**c>C && d<D
**then** 10:          $P^{seg}=P^{seg}-P_{k}^{seg}$ 11:        **end if** 12:   **end for** 13: **end for** 14: $P^{seg}\to P^{sta}$

Let $P^{seg}=\left\{P_{1}^{seg},P_{2}^{seg},...,P_{n}^{seg}\right\}$ be the segmented points from a LiDAR sweep, where $P_{i}^{seg}$ is a subset of the point clouds with the same segmentation label *i*. Let $P^{rad}=\left\{p_{1}^{rad},p_{2}^{rad},...,p_{n}^{rad}\right\}$ be the detected targets after velocity compensation by mmW-radar. $P^{seg}$ and $P^{rad}$ are the inputs of the algorithm. First, the LiDAR point cloud $P^{seg}$ is projected onto the x-y plane, denoted by $P^{seg^{\prime }}=\left\{P_{1}^{seg^{\prime }},P_{2}^{seg^{\prime }},...,P_{n}^{seg^{\prime }}\right\}$, and stored in a KD tree. For each radar target $p_{j}^{rad}$, the LiDAR points neighbors around it within radius $R_{search}$ are searched and collected as a point cloud $P^{nb}$. Then we analyze all the points in $P^{nb}$. Let $P_{k}^{nb}$ be the points with label *k* within $P^{nb}$. If the number of points within $P_{k}^{nb}$ is above the threshold *C* and the distance between centroid of segmentation $P_{k}^{seg^{\prime }}$ and the radar point is below the threshold *D*, then the segmented LiDAR points $P_{k}^{seg}$ is considered to be some correspondence to the radar target $p_{j}^{rad}$, i.e., a moving target, and then all the points labeled with *k* will be removed from the point clouds. Finally, the algorithm outputs a filtered point cloud $P^{sta}$ corresponding to the environment excluding any moving object.

**Figure 7:**
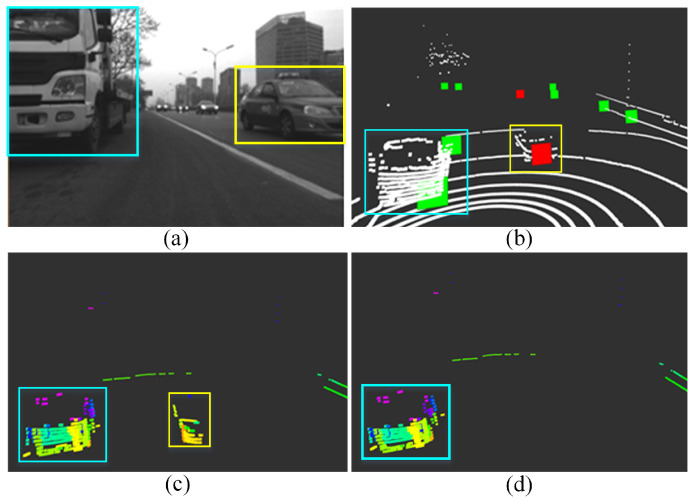
Moving targets detection and removal test. (**a**) Test scenario, two targets are detected by radar. (**b**) Stationary truck (green) and moving cars (red) are distinguished by radar after velocity compensation. (**c**) Segmentation result of point cloud. (**d**) Filtered point cloud.

As shown in Figure 7a, there are a stationary truck and several moving cars on the road. Although both the truck and car are detected as moving targets by radar, by velocity compensation the stationary truck and moving cars can be precisely distinguished, as shown in Figure 7b. In addition, finally the actually moving car is removed from the point clouds as Figure 7d by data association. The proposed data association method can effectively detect the relatively moving objects, and with velocity compensation the absolutely moving objects can be further identified and removed. From the test, the feasibility and accuracy of data association between two sensors with different resolutions are proved.

## 4. Experiments and Results

**Figure 8:**
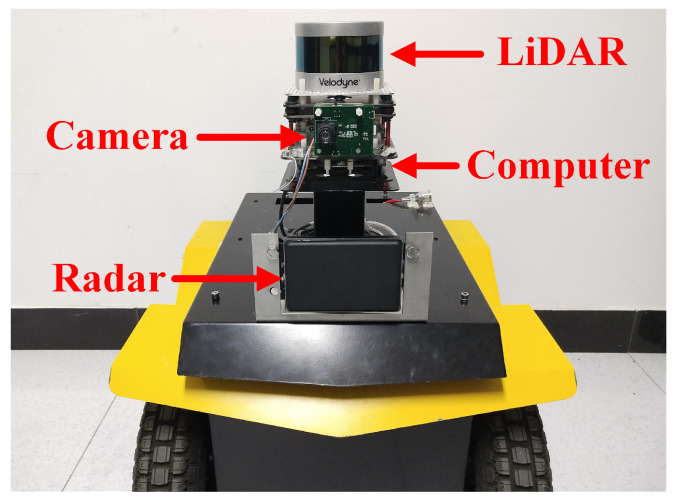
The wheeled robot used in our experiments, equipped with a LiDAR, a mmW-radar, a camera and a computer.

The datasets used for the experiments include open-source KITTI [45] datasets and custom datasets collected using a wheeled robot equipped with a LiDAR (VLP-16) and the mmW-radar (Delphi ESR), as shown in Figure 8. VLP-16 is a 16-channel range finder with detection range of 100 m and FOV of $30^{\circ }\times 360^{\circ }$. The radar provides two measurement modes simultaneously, including a wide FOV of $90^{\circ }$ at mid-range and a small FOV of $20^{\circ }$ at long-range, but only outputs 2D position of the targets in horizontal scanning plane. A camera is mounted to record the scenario. All the algorithms are tested on an Intel NUC computer (i7-5557U CPU @ 3.10 GHz and 8.0 GB RAM) with ROS.

We conducted a series of experiments using two open-source algorithm, LOAM [9] and LeGO-LOAM [43]. Both algorithms can realize localization and 3D mapping based on LiDAR solely. We use these algorithms as a baseline to evaluate the influence of moving objects in the performance of LiDAR-SLAM, and demonstrate the evident improvement of the proposed moving object elimination method.

### 4.1. Evaluation of the State-of-the-Art LiDAR SLAMs in Dynamic Environments

In this section, we evaluate the performance of LiDAR-based SLAM in various scenarios with different dynamic patterns through semi-physical simulation. To quantitatively analyze the results, we calculate the root mean square error (RMSE) of the absolute position error in meters, representing the accuracy performance of the algorithm. RMSE is defined in Equation (9).

(9) $$RMSE=\sqrt{\frac{1}{n}\sum_{i=1}^{n}{\left(\overset{\wedge }{p_{i}}-p_{i}\right)}^{2}}$$

where *n* is the number of estimates, $\overset{\wedge }{p_{i}}=\left(\overset{\wedge }{x_{i}},\overset{\wedge }{y_{i}},\overset{\wedge }{z_{i}}\right)$ is the *i*th estimate, and $p_{i}=\left(x_{i},y_{i},z_{i}\right)$ is the corresponding ground truth.

**Figure 9:**
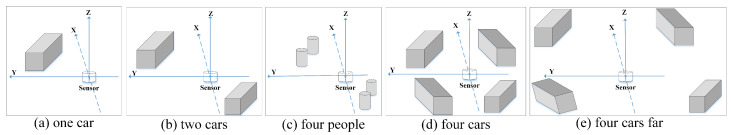
The diagram of the five different dynamic patterns used in semi-physical simulation. By comparing (**a**,**b**,**d**), we can analyze the influence of number of dynamic objects on performance of SLAM. By comparing (**b**,**c**), we can analyze the impact of shape of the objects on SLAM performance. The influence of distribution on SLAM can be obtained by comparing (**d**,**e**).

**Figure 10:**
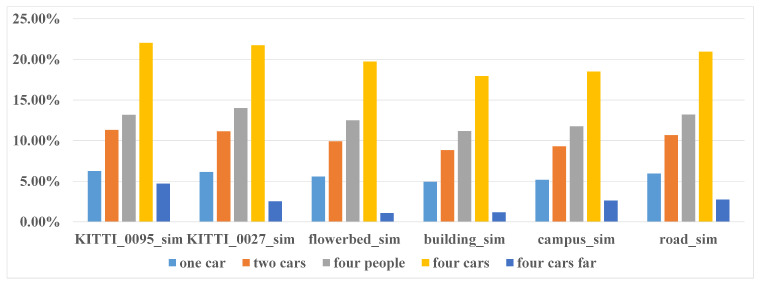
The moving objects proportion of the five scenarios in the six datasets of semi-physical simulation.

To conduct a comprehensive analysis, we simulated five different dynamic patterns based on six real datasets, totally got 30 semi-physical simulated datasets. The first four dynamic patterns are: one car, two cars, four people, four cars moving in the environment but relatively static with the robot. In the fifth pattern four cars are moving in the environment with random speed and relatively moving with the robot. The diagram of the five different dynamic patterns is shown in Figure 9. By comparing (a), (b) and (d), we can analyze the influence of number of dynamic objects on performance of SLAM. By comparing (b) and (c), we can analyze the impact of shape of the objects on SLAM performance. The influence of distribution on SLAM can be obtained by comparing (d) and (e). Through our analysis, we found that both the quantity and the distribution are closely related to the proportion of the moving objects in the total point cloud. Therefore, we evaluate the SLAM performance directly with respect to the proportion of the moving objects that calculated by Equation (10). The six real datasets used here include two KITTI datasets (sequence 2011_09_26_0095 and 2011_09_30_0027) and four custom datasets (flowerbed, research buildings, campus and road).

(10) $$ratio=\frac{1}{n}\sum_{i=1}^{n}\frac{N\_sim}{N\_sum}$$

where *n* is the number of LiDAR sweep, N_sim is the point number of simulated moving objects and N_sum is total number of points in this sweep. The proportion of moving objects in the five scenarios of six datasets are shown in Figure 10.

#### 4.1.1. Evaluation on KITTI Datasets

**Figure 11:**
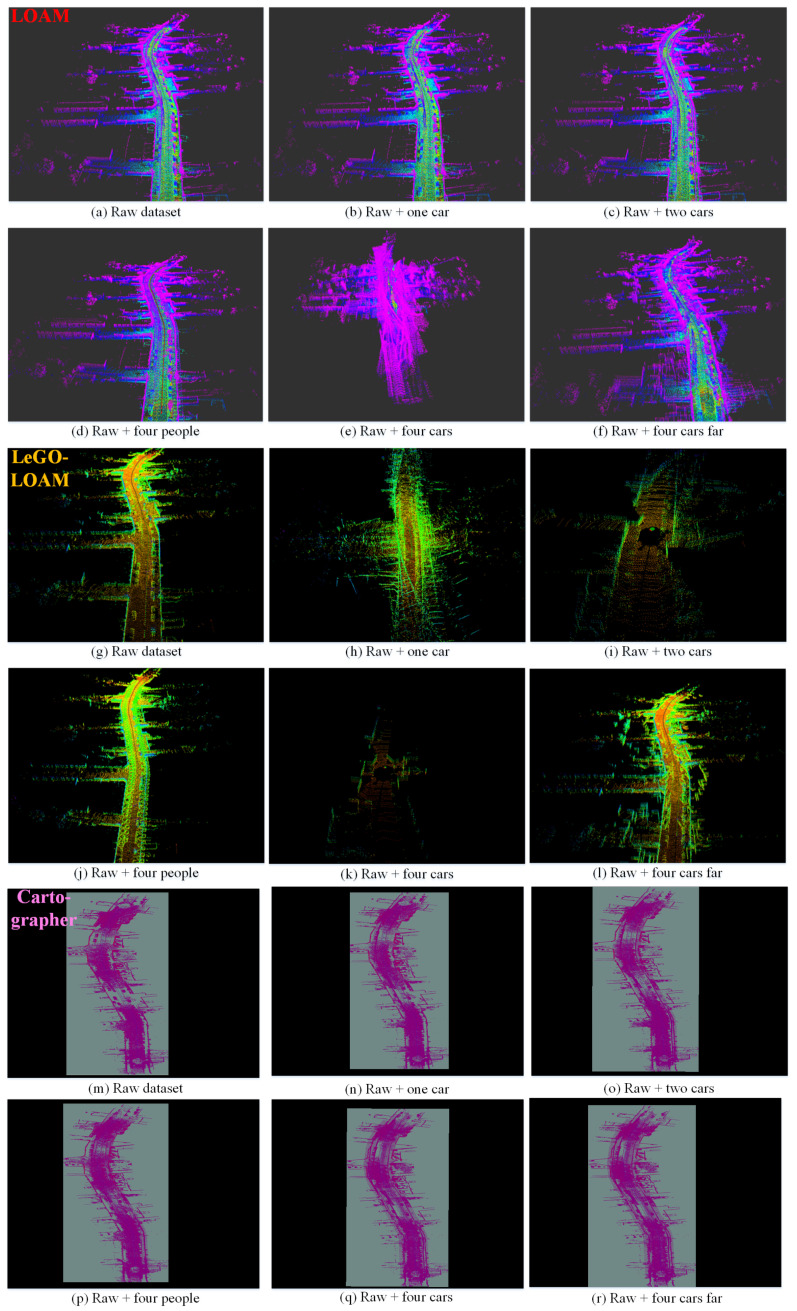
The mapping results (3D view) of LOAM (**a**–**f**), LeGO-LOAM (**g**–**l**) and Cartographer (**m**–**r**) in different simulated scenarios based on KITTI_0095_sim. (**a**,**g**,**m**) are the results on the original static data, used as baseline. More dynamic objects result in worse mapping results, and even total failure in some scenarios such as (**e**,**h**,**i**,**k**). LeGO-LOAM is more sensitive to moving objects in this test.

**Figure 12:**
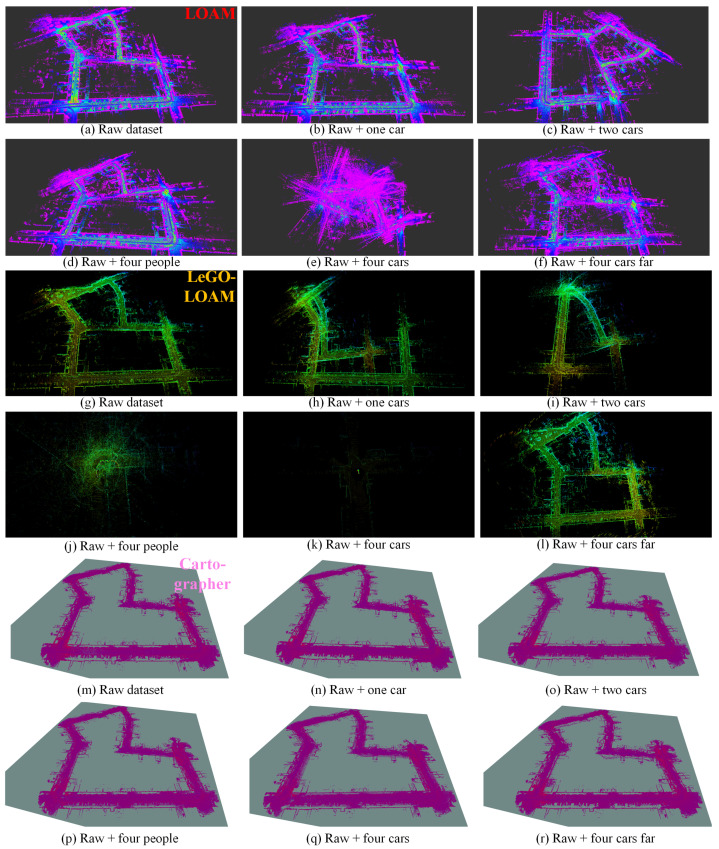
The mapping results (3D view) of LOAM (**a**–**f**), LeGO-LOAM (**g**–**l**) and Cartographer (**m**–**r**) in different simulated scenarios based on KITTI_0027_sim. (**a**,**g**,**m**) are the results on the original static data, used as baseline. Please note that in (**c**) estimation error occurs at the beginning, thus results in the deflected map. More dynamic objects result in worse mapping results, and even total failure in some scenarios such as (**e**,**h**,**i**–**k**). In this test Cartographer shows relatively more robust performance.

**Figure 13:**
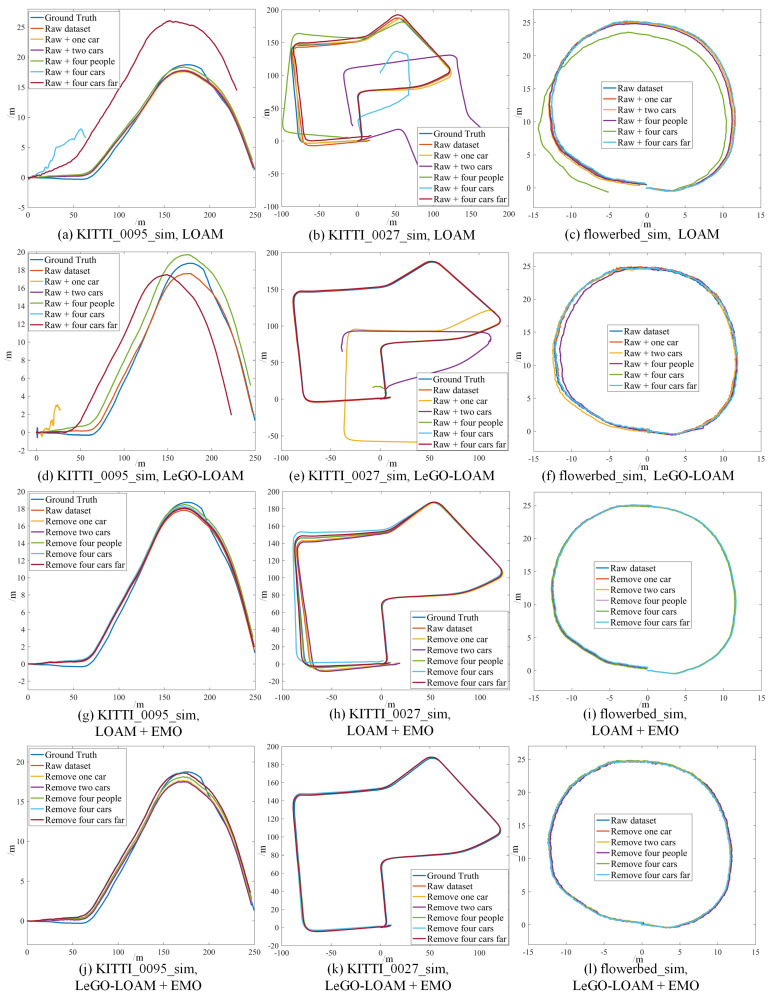
The odometry results of [original LOAM] (**a**–**c**), [original LeGO-LOAM] (**d**–**f**), [LOAM + EMO] (**g**–**i**) and [LeGO-LOAM + EMO] (**j**–**l**) in different simulated scenarios based on KITTI_0095_sim, KITTI_0027_sim and flowerbed_sim. Before removing the dynamic objects, more dynamic objects result in worse odometry drift or even estimation failure (**a**–**f**). After removing the moving objects with our method, the two algorithms yield evidently improved odometry accuracy in all the scenarios (**g**–**l**).

**Figure 14:**
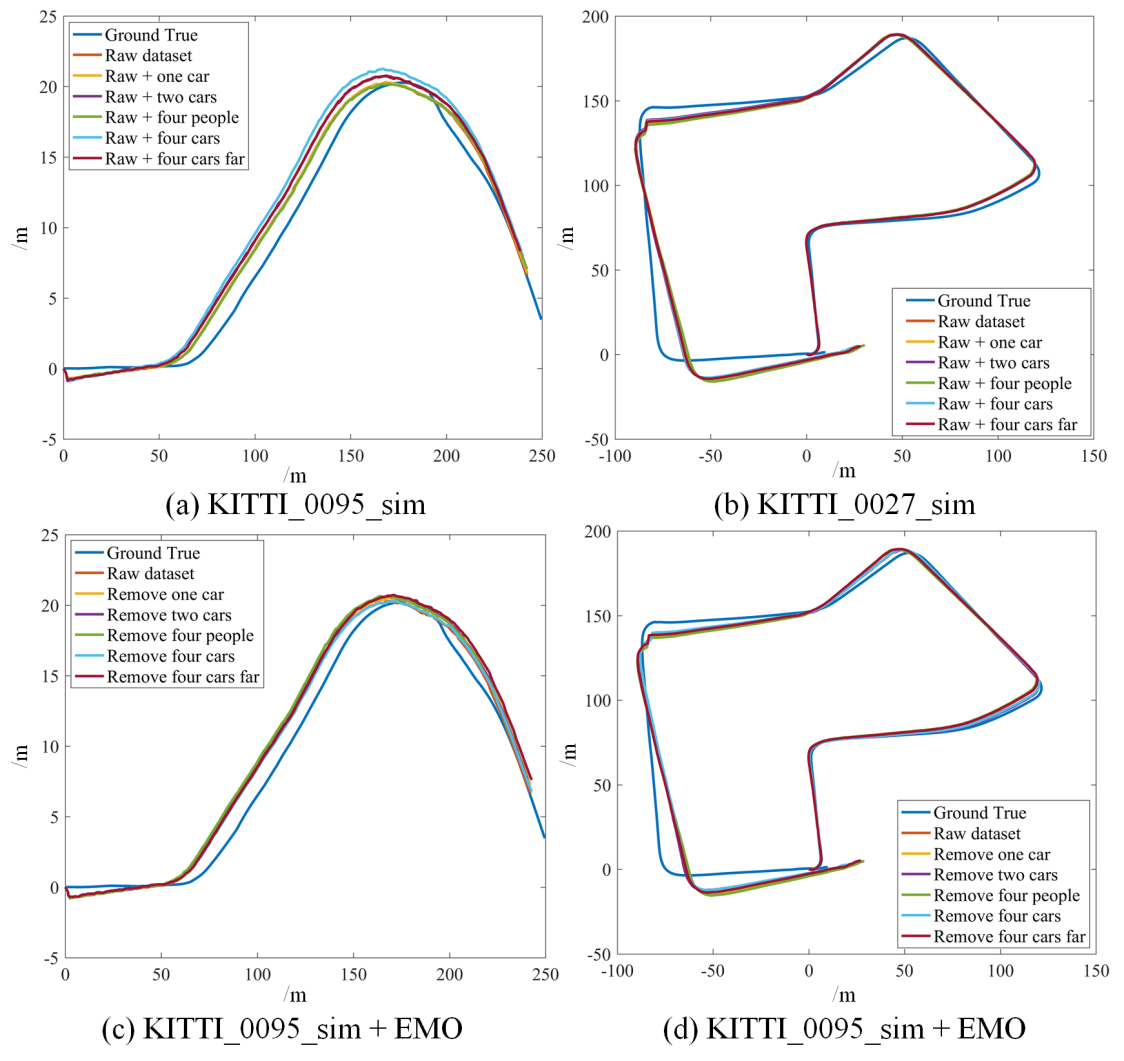
The odometry results of [Cartographer] (**a**,**b**) and [Cartographer + EMO] (**c**,**d**) based on KITTI_0095_sim and KITTI_0027_sim. After removing the moving objects with our method, it yields evidently improved odometry accuracy in all the scenarios.

We choose two sequences from KITTI datasets, KITTI 2011_09_26_0095 and KITTI 2011_09_30_0027 since there are few moving objects in these original datasets; thus we can simulate the dynamic targets based on the static environment. We test Cartographer, LOAM and LeGO-LOAM on the semi-physically simulated data. The mapping results are shown in Figure 11 and Figure 12 and the odometry results are shown in Figure 13a,b,d,e and Figure 14. Basically, more objects will result in worse estimation result, including large odometry drift and mapping inconsistency. In particular, compared with pedestrian we found that cars have much bigger influence on the mapping performance, and even result in total failure in some scenarios.

Furtherly we compute RMSE of the absolute position error for quantitative evaluation of the odometry results, as shown in the first two rows of Table 1. The bold number indicate obvious odometry drift or failure. From the result, we can see that more moving objects will cause worse estimation results. Again, we can notice that different object models have different effects on the performance, i.e., LOAM and LeGO-LOAM are more sensitive to the cars than pedestrian. The reason for this phenomenon is that we use cuboid to model the car and it provides intense edge and plain structure features to the algorithm, which are just the feature information used in LOAM and LeGO-LOAM. On the contrary, the cylinder model for pedestrian perform smaller effect in this aspect.

#### 4.1.2. Evaluation on our Datasets

**Figure 15:**
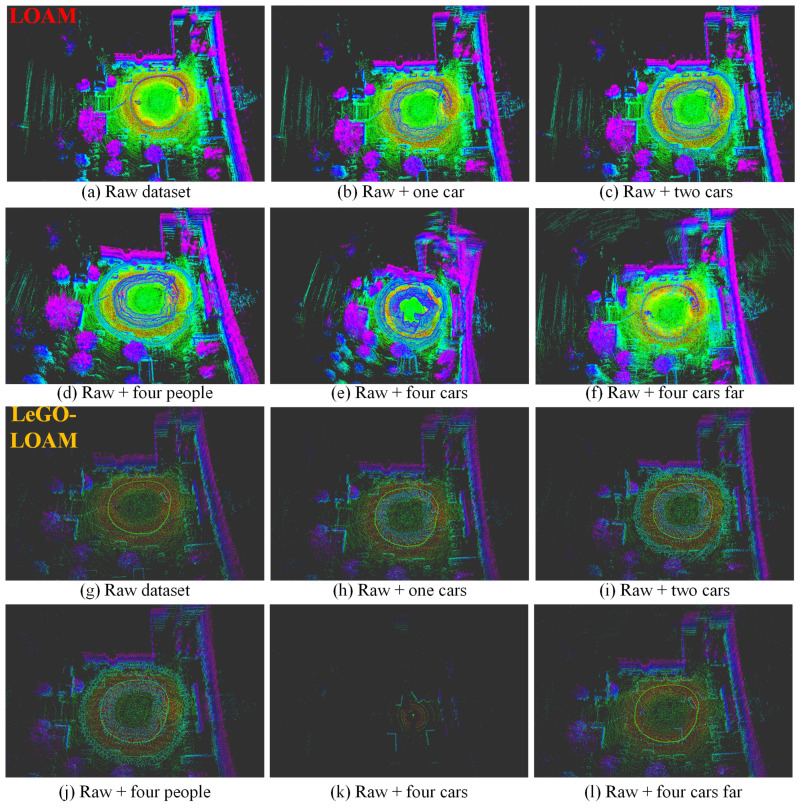
The mapping results (3D view) of LOAM (**a**–**f**) and LeGO-LOAM (**g**–**l**) in different simulated scenarios based on flowerbed_sim. (**a**,**g**) are the results on the original static data, used as baseline. More dynamic objects result in more obvious mapping error such as (**e**), or even total failure in some scenarios such as (**k**).

We collected a set of datasets using wheeled robot in static environment and use the same semi-physical simulation method to generate four datasets with different dynamic scenarios: flowerbed, building, campus, road. The odometry and mapping results of LOAM and LeGO-LOAM on dataset flowerbed are shown in Figure 13c,f and Figure 15. The odometry of both algorithms drift to some degree when moving objects exist in the environment. Due to limited space, the results of datasets building, campus and roads are given quantitatively only, in the last four rows of Table 1.

The quantitative analysis results of RMSE of position error are shown in the last four rows of Table 1. Please note that the ground truth is not available in our datasets, so we use the odometry results of baseline, i.e., the result on the raw data without dynamic objects as ground truth to compute RMSE. The obvious performance degradation or failure is indicated in bold. We can clearly see that more moving objects in the environment will cause worse estimation result and both algorithms are more sensitive to the cars because cuboids provide intense edge and plain features to the algorithms.

Through the semi-physical simulation-based experiments with different scenarios in different datasets, we can draw the following conclusions: (1) The presence of dynamic objects does affect the perceptual positioning and mapping performance of robots, and this effect is related to many factors such as the shape, quantity, speed and distribution of moving objects. (2) For the same type of moving objects, the more the proportion of the moving objects in the LiDAR sweep is, the worse the estimation result of odometry and map will be. The proportion is affected by the quantity and distribution (distance) of the objects. (3) For the different types of moving objects, LOAM and LeGO-LOAM are more sensative to the cars that modeled using cuboid, which means moving cars can introduce more estimation error than pedestrian. This is because cuboid provide intense edge and plain features, which is just LOAM and LeGO-LOAM used for scan matching. For Cartographer, we found that cars and pedestrians perform similar influence on the estimation result because Cartographer uses a grid map-based method in which all the points are considered instead of feature points only. (4) The objects’ speed can also make a difference. When the moving objects remain relatively static with the robot, the influence of the dynamic objects is greater.

### 4.2. LiDAR-SLAM with Proposed Moving Objects Elimination

In this section, we evaluate and demonstrate the performance improvement of LiDAR-SLAM with our proposed moving objects elimination method, using both semi-physically simulated datasets and real-world datasets.

#### 4.2.1. On Semi-Physically Simulated Datasets

Semi-physically simulated datasets are used here to verify the effectiveness of the proposed method. As mmW-radar is used in the elimination stage, the radar-detected targets are also required in the datasets. Due to the position of the simulated moving object is known when we generate the dataset, accordingly we simulate the mmW-radar targets with specified noise at the same time. Thus, the datasets including point clouds and radar targets are ready for the experiments.

In the proposed method, the point clouds are segmented and moving targets are detected and verified using LiDAR and radar, then the point clouds corresponding to the dynamic objects are removed and filtered point clouds are used as the input of the LOAM and LeGO-LOAM. The odometry results of LOAM and LeGO-LOAM on two KITTI datasets and one custom dataset are depicted in Figure 13g–l to representatively show the effectiveness of our method. Compared with Figure 13a–f we can clearly see the significant improvement on the accuracy of the state estimation for all the six scenarios in the three datasets. Consequently, the mapping results are also precise, and will not be shown in this paper.

Also, we quantitatively analyzed the position error for all the six datasets, as shown in Table 1. Compared with Table 1, the absolute position error is significantly reduced even in highly dynamic environment, which provides a solid evidence of the effectiveness of the proposed method.

#### 4.2.2. On Real-World Dynamic Datasets

We further demonstrate the proposed method using three real-world datasets collected in dynamic environments with our wheeled robot. In particular, we equipped the robot with a high-precision inertial and GNSS-based navigation system to get the ground truth in these experiments.

**Figure 16:**
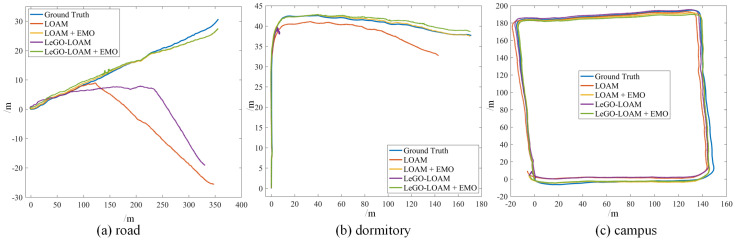
The odometry results of three datasets: straight road (**a**), dormitory park (**b**) and campus (**c**).

**Figure 17:**
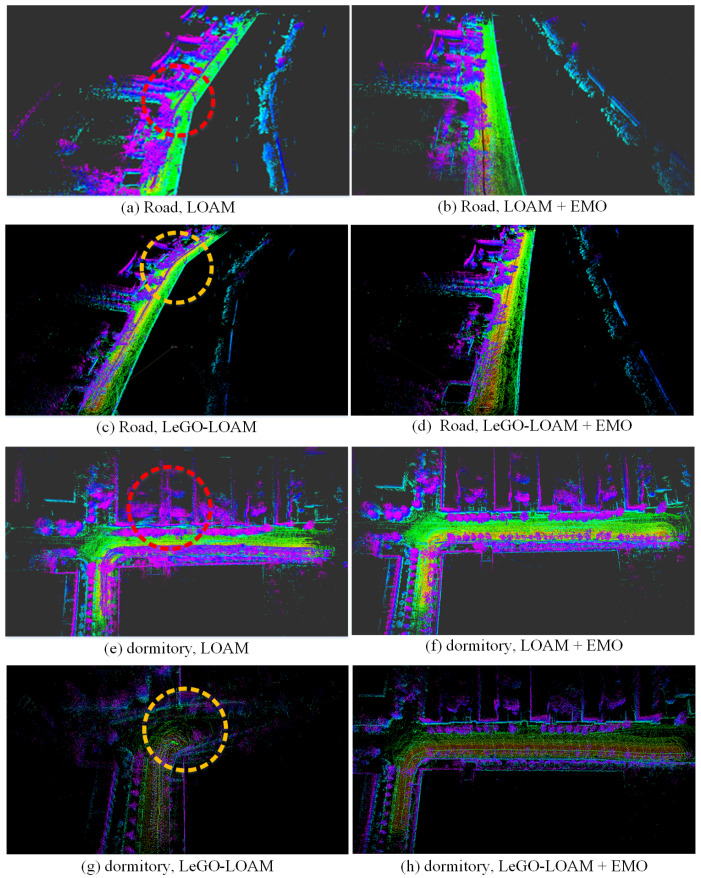
The mapping results (3D view) of dataset road (**a**–**d**) and dormitory (**e**–**h**). (**a**,**c**,**e**,**g**) are the maps built by LOAM and LeGO-LOAM before removing dynamic objects, in which the significant mapping errors are marked using circles. (**b**,**d**,**f**,**h**) are the improved mapping results using our method (EMO) of moving object elimination, showing better map consistency and sharper detail.

In the first test, the robot moves along a side road with some vehicles and pedestrians moving on the road. We compare the odometry and mapping results of LOAM and LeGO-LOAM with and without the moving objects elimination, as shown in Figure 16a and Figure 17a–d. From the results it can be seen that before removing dynamic targets the odometry seriously diverged from the ground truth. The moving objects made an effect on the inter-frame registration, which leads to the positioning error and consequential mapping degradation. However, after removing dynamic objects using our method the estimator yields remarkable improvement including pose accuracy (position error decreased by at least 84.9%) and mapping consistency.

In the second test, the robot moves in a dormitory park with several rows of buildings, with some pedestrians walking around. The results of odometry and mapping are depicted in Figure 16b and Figure 17e–h. Before removing dynamic objects, LeGO-LOAM totally failed. With our method, the degradation of state estimation is significantly mitigated (position error decreased by at least 90%).

In the last test we extend our experiment in a large scenario. The robot moves around a campus with a few of moving targets and returns to the starting point. The odometry result is shown in Figure 16c. By investigating the quantitative result in Table 2, we can still tell the minor improvement of accuracy (position error decreased by at least 30% after using our method, though there only exist a few dynamic objects in the experiment.

The three corresponding quantitative results expressing the position RMSE are shown in Table 2, in which the positioning error significantly decreased (position error decreased by at least 30%) after using our method to remove the dynamic objects.

From the simulated and real-world experiments, we can clearly see that benefiting from the well-designed sensor fusion and data association, the proposed method can effectively improve the performance of the LiDAR-based SLAM on accuracy and robustness even in highly dynamic environments, without increasing computation significantly.

## 5. Conclusions

The existence of moving objects will affect the accuracy of localization and consistency of mapping to varying degrees, and subsequently degrade the reliability of autonomous vehicles. To quantitatively analyze the impact of dynamic objects on the state-of-the-art SLAM performance, we propose a semi-physical simulation method to generate datasets with different dynamic patterns by integrating real-world static data and simulated dynamic data. From the analysis result we found that the impact of dynamic environment is related to the shape, number, speed and distribution of moving objects, which provide a theoretical an empirical background for the following proposed moving object elimination method.

Benefiting from the complementary characteristics of mmW-radar and LiDAR, we propose a method to detect, identify and eliminate efficiently and precisely the moving objects by well-designed sensor fusion and data association, towards enhancing the performance of dynamic SLAM in the real world. At the same time, the temporal synchronization and spatial calibration between the sensors are carefully designed to ensure effective sensor fusion. Through the semi-physical simulation and real-world experiments the effectiveness and performance of the proposed method are demonstrated and evaluated, showing significant improvement in the odometry accuracy (position error decreased by at least 30%), mapping consistency and estimation robustness, without increasing much computing. The proposed method can also be applied to space robots, in orbital or on planetary environments, in which multi-agent robots, astronauts, and other floating objects may exist and form a challenging dynamic scenario.

The focus of this paper is to fuse the information of the two sensors at the front end to remove the dynamic target, and then use the filtered point cloud as the input of LiDAR-SLAM to improve the overall performance, which is a loosely coupled method to fuse the LiDAR and mmW-radar. To fully take the advantage of laser and microwave sensors, our future work involves integrating high-resolution 3D mmW-radar and developing a tightly coupled SLAM using LiDAR and mmW-radar to further improve the robustness and accuracy of localization and mapping. For example, the information of the reflection intensity provided by the radar can be used in frame matching and provide more stable constraints in global optimization to get better results.

## Figures and Tables

**Table 1:** The absolute position RMSE (unit: m) of odometry results from Cartographer, LOAM and LeGO-LOAM in six semi-physically simulated datasets with and without our proposed method (EMO). The bold number indicate obvious odometry drift or failure.

Datasets	Algorithm	One Car	Two Cars	Four People	Four Cars	Four Cars Far
KITTI_0095_sim	LOAM	0.5047	0.8424	0.7843	**108.8954**	**19.7693**
	LOAM + EMO	0.3430	0.3618	0.2680	0.4940	0.2551
	LeGO-LOAM	**126.4614**	**140.5702**	3.0814	**141.1223**	**23.4166**
	LeGO-LOAM + EMO	0.1271	0.1973	0.4182	1.9146	1.7046
	Cartographer	0.9408	2.0061	1.8029	2.7668	1.4945
	Cartographer + EMO	0.8899	0.9732	1.1103	1.2149	1.3603
KITTI_0027_sim	LOAM	4.9643	**145.0990**	19.3609	**109.1039**	6.7535
	LOAM + EMO	2.1389	3.8326	2.7545	5.8555	2.6443
	LeGO-LOAM	**59.9155**	**112.3563**	**117.0891**	**125.8396**	1.3541
	LeGO-LOAM + EMO	0.7182	1.1032	0.8759	1.4460	0.3776
	Cartographer	2.3954	2.4832	3.4827	2.5367	1.1656
	Cartographer + EMO	1.7730	2.3029	2.7699	2.1686	1.1010
flowerbed_sim	LOAM	0.3081	0.6780	0.5914	**3.0507**	0.2593
	LOAM + EMO	0.3079	0.3760	0.1974	0.5848	0.2689
	LeGO-LOAM	1.1677	**2.2230**	1.3041	**16.3797**	0.4063
	LeGO-LOAM + EMO	0.3416	0.4321	0.3888	0.2391	0.0845
building_sim	LOAM	1.2965	1.9941	1.4872	**14.5741**	1.8203
	LOAM + EMO	0.5387	1.2813	1.1456	0.8995	1.3091
	LeGO-LOAM	1.9142	**8.0904**	3.1911	**34.2463**	1.7784
	LeGO-LOAM + EMO	0.5049	0.9626	0.7467	1.7590	0.4377
campus_sim	LOAM	0.5859	0.8080	0.6600	**6.2356**	0.2192
	LOAM + EMO	0.0698	0.1199	0.0790	0.2645	0.0337
	LeGO-LOAM	0.4945	**21.3248**	**21.1645**	**21.7350**	0.1855
	LeGO-LOAM + EMO	0.0812	0.1069	0.1419	0.2419	0.1569
road_sim	LOAM	0.5011	**8.2676**	**7.8464**	**41.0379**	2.1865
	LOAM + EMO	0.4941	0.5275	0.4359	4.7984	0.1683
	LeGO-LOAM	6.8698	**25.1214**	**19.1836**	**44.0201**	5.1829
	LeGO-LOAM + EMO	0.1534	4.9771	4.7344	5.1979	4.9042

**Table 2:** The position RMSE (unit: m) of LOAM and LeGO-LOAM in three real-world dynamic tests with and without the moving objects elimination (EMO). The bold number indicate obvious improvement and smaller error. % indicates the percentage of error reduction.

	LOAM	LOAM + EMO	LeGO-LOAM	LeGO-LOAM + EMO
**Road**	111.7965	**3.9360** (96.48%)	29.5669	**4.4634** (84.90%)
**Dormitory**	15.2370	**1.3472** (91.16%)	80.5292	**0.9339** (98.84%)
**Campus**	5.7304	**3.0851** (46.16%)	4.9835	**3.5135** (29.50%)
